# Draft Genome Sequence of *Planomicrobium glaciei* UCD-HAM (Phylum *Firmicutes*)

**DOI:** 10.1128/genomeA.01209-15

**Published:** 2015-10-15

**Authors:** Makayla N. Betts, Guillaume Jospin, Jonathan A. Eisen, David A. Coil

**Affiliations:** a Davis Genome Center, University of California, Davis, California, USA; b Department of Evolution and Ecology, University of California Davis, Department of Medical Microbiology and Immunology, Davis, California, USA

## Abstract

Here, we present the draft genome of *Planomicrobium glaciei*, a member of the phylum *Firmicutes*, found at the University of California Davis. Paired-end, 300-bp reads were generated on an Illumina MiSeq. The assembly consists of 3,925,122 bp, contained in 109 contigs, with a G+C content of 46.7%.

## GENOME ANNOUNCEMENT

The *Planomicrobium* genus, proposed by Yoon et al. in 2001, characterizes non-spore-forming, aerobic bacteria that produce yellow-orange pigmented colonies and have a DNA G+C content between 35% and 47% (1). The psychrotolerant species *Planomicrobium glaciei* was established based on an isolate from the frozen soil of a Chinese glacier in 2009 (2). The isolate presented here was cultured from the fabric of an outdoor hammock at the University of California, Davis.

The hammock was swabbed with a dry sterile cotton swab, which was then streaked and incubated aerobically on a lysogeny broth (LB) agar plate at room temperature for 3 days. Subsequent isolation (double-dilution streaking) yielded convex orange colonies with circular, even margins. Genomic DNA was extracted using the Wizard genomic DNA purification kit (Promega). Paired-end libraries were made using the Nextera kit by Illumina.

A total of 3,924,164 paired-end reads were generated on an Illumina MiSeq, at a read length of 300 bp. Quality trimming and error correction of the reads resulted in 3,865,931 high-quality reads. All sequence processing and assembly was performed using the A5 assembly pipeline (3). This pipeline automates the processes of error correction, data cleaning, contig assembly, and quality control. The resulting assembly produced 109 contigs, with an *N*_50_ of 161,194. The resulting genome consisted of 3,925,122 bp with a GC content of 46.7% and an overall coverage estimate of 250×. Completeness of the genome was assessed using the PhyloSift software (4), which searches for a list of 37 highly conserved, single-copy marker genes (5), all of which were found in this assembly.

Automated annotation was performed using the Rapid Annotation using Subsystems Technology (RAST) server (6). The isolate’s 16S rRNA gene sequence matched with the species *Planomicrobium glaciei* with 99% identity and an E value of 0.0 on NCBI’s BLAST (7). A phylogenetic tree containing multiple *Planomicrobium* species confirmed that this isolate occurred in a monophyletic clade of *Planomicrobium glaciei* (tree available at http://dx.doi.org/10.6084/m9.figshare.1532940). *Planomicrobium glaciei* UCD-HAM contains 3,864 predicted protein-coding genes and 89 predicted noncoding RNAs. A comparison to the RAST annotation of the isolate from the Chandra River, *Planomicrobium glaciei* CHR43 (8), revealed the two strains to be markedly similar, predicting similar metabolic capacities. The most prominent difference was that the UCD-HAM strain has 37 genes predicted for flagellar motility, while the CHR43 strain has none.

### Nucleotide sequence accession numbers.

This whole-genome shotgun project has been deposited at DDBJ/EMBL/GenBank under the accession number LGAF00000000. The version described in this paper is version LGAF01000000.

## References

[1] Yoon JH, Kang SS, Lee KC, Lee ES, Kho YH, Kang KH, Park YH 2001 *Planomicrobium koreense* gen. nov., sp. nov., a bacterium isolated from the Korean traditional fermented seafood jeotgal, and transfer of *Planococcus okeanokoites* (Nakagawa et al. 1996) and *Planococcus mcmeekinii* (Junge et al. 1998) to the genus *Planomicrobium*. Int J Syst Evol Microbiol 51:1511–1520. doi:10.1099/00207713-51-4-1511. 11491353

[2] Zhang DC, Liu HC, Xin YH, Yu Y, Zhou PJ, Zhou YG 2009 *Planomicrobium glaciei* sp. nov., a psychrotolerant bacterium isolated from a glacier. Int J Syst Evol Microbiol 59:1387–1390. doi:10.1099/ijs.0.002592-0. 19502321

[3] Tritt A, Eisen JA, Facciotti MT, Darling AE 2012 An integrated pipeline for *de novo* assembly of microbial genomes. PLoS One 7:e42304. doi:10.1371/journal.pone.0042304. 23028432 PMC3441570

[4] Darling AE, Jospin G, Lowe E, Matsen FA, Bik HM, Eisen JA 2014 PhyloSift: phylogenetic analysis of genomes and metagenomes. PeerJ 2:e243. doi:10.7717/peerj.243. 24482762 PMC3897386

[5] Wu D, Jospin G, Eisen JA 2013 Systematic identification of gene families for use as “markers” for phylogenetic and phylogeny-driven ecological studies of *Bacteria* and *Archaea* and their major subgroups. PLoS One 8:e77033. doi:10.1371/journal.pone.0077033. 24146954 PMC3798382

[6] Aziz RK, Bartels D, Best AA, DeJongh M, Disz T, Edwards RA, Formsma K, Gerdes S, Glass EM, Kubal M, Meyer F, Olsen GJ, Olson R, Osterman AL, Overbeek RA, McNeil LK, Paarmann D, Paczian T, Parrello B, Pusch GD, Reich C, Stevens R, Vassieva O, Vonstein V, Wilke A, Zagnitko O 2008 The RAST server: rapid annotations using subsystems technology. BMC Genomics 9:75. doi:10.1186/1471-2164-9-75. 18261238 PMC2265698

[7] Altschul SF, Gish W, Miller W, Myers EW, Lipman DJ 1990 Basic local alignment search tool. J Mol Biol 215:403–410. doi:10.1016/S0022-2836(05)80360-2. 2231712

[8] Salwan R, Swarnkar MK, Singh AK, Kasana RC 2014 First draft genome sequence of a member of the genus *Planomicrobium*, isolated from the Chandra River, India. Genome Announc 2(1):e01259-13. doi:10.1128/genomeA.01259-13. PMC3916493 24503999

